# The IL-23p19/EBI3 heterodimeric cytokine termed IL-39 remains a theoretical cytokine in man

**DOI:** 10.1007/s00011-019-01235-x

**Published:** 2019-04-13

**Authors:** Charlie Bridgewood, Adewonuola Alase, Abdulla Watad, Miriam Wittmann, Richard Cuthbert, Dennis McGonagle

**Affiliations:** 1 Leeds Institute of Rheumatic and Musculoskeletal Medicine (LIRMM), University of Leeds, Leeds, UK; 2 Department of Medicine “B”, Zabludowicz Center for Autoimmune Diseases, Sheba Medical Center, Tel-Hashomer, Ramat Gan, Israel; 3 Sackler Faculty of Medicine, Tel-Aviv University, Tel Aviv, Israel; 4 National Institute for Health Research (NIHR) Leeds Biomedical Research Centre (BRC), Leeds Teaching Hospitals, Leeds, UK

**Keywords:** Cytokine, IL-23, Lupus, Psoriasis, IL-39

## Abstract

**Objective:**

The heterodimeric IL-12 family member cytokines including, IL-12, IL-23, IL-27, and IL-35 and have multiple roles in regulating innate and adaptive immunity with crucial functions in inflammatory disorders such as psoriasis. Chain pairing promiscuity is a feature of the IL-12 family. Recently, based on murine data, a new family member, IL-39, was proposed, consisting of IL23p19 (shared with IL-23) and EBI3 (shared with IL-27 and IL-35). IL-39 has subsequently been implicated in experimental murine lupus. Given the success of IL-23p19 therapeutic targeting in diseases including psoriasis, it is of great interest to confirm the presence of IL-39 in man. Human IL-39 is yet to be either detected or expressed, which has halted research in this area.

**Methods:**

Using a disulphide-linked human chimera protein composing of IL-23p19 and EBI3 human chains, we stimulated human leukocytes, and analysed cytokine secretion and STAT3 phosphorylation.

**Results and Conclusion:**

We report that this cytokine shows no activity in human cells. IL-39 chimera protein failed to induce either IL-6, IL-8, TNF, or IL-17A from leukocytes or STAT3 phosphorylation and thus, remains a ‘theoretical cytokine' in humans.

## Introduction

Cytokines are usually grouped into families, which are based on amino acid homology and structural characteristics. The IL-12 family members are unique, in that they form heterodimers comprised of α-subunits and β-subunits [1]. IL-12 was the first member of this family to be discovered, and along with other family members, IL-23 and IL-27, they are predominantly secreted by activated antigen presenting cells [1]. IL-12 has a central role in polarization and promotion of type I immune responses which are characterized by preferential release of interferon gamma [1]. IL-23 induces IL-17 from a range of conventional and non-conventional lymphocytes (the IL-23/IL-17 axis) [2]. While IL-27 shows immune activating activity, the overall (albeit context specific) effect of IL-27 seems to be regulatory in a range of disease models [3]. As recently published [4], the property of IL-27 to act on co-inhibitory receptor regulation seems important. The most recent ‘fully recognized’ member of the family in humans is IL-35. IL-35 is secreted by Tregs and also activated B cells, and has immunosuppressive functions [5, 6]. IL-12 family cytokines have an essential function in a wide range of physiologic responses in particular pathogen defense and protection of the intestinal mucosa and elicit functions via phosphorylation of members of the signal transducer and activator of transcription (STAT) family of transcription factors [1]. As evidenced by the success of IL-12 family-related biologics (ustekinumab, guselkumab, and risankizumab) but also by described pathologies for loss of function mutations of IL-12 cytokine receptors such as Mendelian susceptibility to mycobacterial diseases (MSMD) [7], IL-12 family cytokines are heavily implicated in pathogen defense but also inflammatory disease. IL-23 has been shown to be a key orchestrator of the psoriatic disease spectrum and therapeutic targeting has proved successful in this disease [2, 8].

Recently, a new IL-12 family member was discovered in mice, IL-39. The IL-39 heterodimer consists of IL-23p19 (shared with IL-23) and EBI3 (shared with IL-27 and IL-35). Murine IL-39 is secreted by activated B cells, and activates neutrophils and was shown to mediate inflammation in lupus like mice [9, 10]. Ustekinumab, originally thought to impact on IL-12p40, is now thought to show its disease modifying activity by IL-23 (IL-12p40 + IL-23p19) inhibition. Recent data from several clinical trials as well as real-life clinical observations strongly suggest that IL-23p19 blockers such as risankizumab and guselkumab are superior to ustekinumab in psoriasis [11]. Of course, any efficacy difference between IL-12p40 blockers and IL-23p19 blockers could also be attributed to blocking of the IL-23p19 chain of IL-39. Thus, confirmation of IL-39 in humans is of great interest. Human IL-39 has not yet been detected or expressed, meaning that research into the area is slow. A recent report studying IL-12 family chain pairings in human cells detected all other IL-12 family pairings, but failed to detect a EBI3 + IL-23p19 combination, raising doubts over the existence of this cytokine in man [12]. We, thus, aimed to study the effect of a disulfide-linked IL-39 chimera protein on human cells.

## Methods

### Leukocyte stimulation and ELISA

Blood from healthy volunteers was collect in EDTA tubes. Red blood cells were lysed with red cell lysis buffer and leukocytes were subsequently plated out at a concentration of 5 × 10^5^ per well in RPMI 1640 +10% FCS. Cells were stimulated with LPS (50 ng/ml) (Sigma) or IL-39 (10–100 ng/ml) (R&D Systems) for 48 h. Secretion of TNF, IL-6, and IL-8 into the supernatant was tested by ELISA. For IL-17 ELISA measurement, as a positive control 5 × 10^5^ cells were stimulated with plate bound anti CD3 (10 µg/ml) and anti-CD28 (10 µg/ml) (both Thermofisher) for 48 h. All ELISA kits were purchased from Thermofisher and carried out according to the manufacturer’s instructions. Analysis was performed using GraphPad Prism software (GraphPad Software Inc, La Jolla, CA, USA). Error bars represent the standard error of the mean (SEM).

### Western blot and flow cytometry

Cells were stimulated as before with IL-39 (100 ng/ml) or IL-6 (20 ng/ml) (Peprotech). Cells were lysed with CelLytic M lysis buffer (Sigma-Aldrich), containing protease inhibitor cocktail (Roche Applied Bioscience, Rotkreuz, Switzerland) and phosphatase inhibitor (Thermofisher). Protein concentration was determined by Bradford Assay, and 30 μg of total protein was separated on any kDa mini protean gel (Bio-Rad). Proteins were blotted onto 0.2 μm PVDF trans-blot pack (Bio-Rad). Membranes were incubated with either mouse anti-human GAPDH (Santa Cruz 1:10,000), mouse anti-human STAT3, or mouse anti-human phospho-STAT3 (both Cell Signaling, 1:1000), in 5% BSA/TBST overnight at 4 °C. Membranes were subsequently incubated with secondary antibody, HRP conjugated donkey anti-mouse (Santa Cruz, 1:5000) for 1 h at room temperature. Substrate solution A and B was from GE healthcare. For flow cytometry analysis, cells were stimulated as before. Using IntraPrep Permeabilizaton kit (Beckman Coulter) according to the manufacturer’s instructions, cells were stained with mouse anti-human phospho-STAT3 or isotype control (PE-Cyanine7, eBioscience, both 1:50). Cells were analysed using the LSRII (BD Biosciences) and a FlowJo software (Tree Star Software, San Carlos, California, USA).

## Results

### IL-39 does not induce cytokine secretion from human leukocytes

**Fig. 1:**
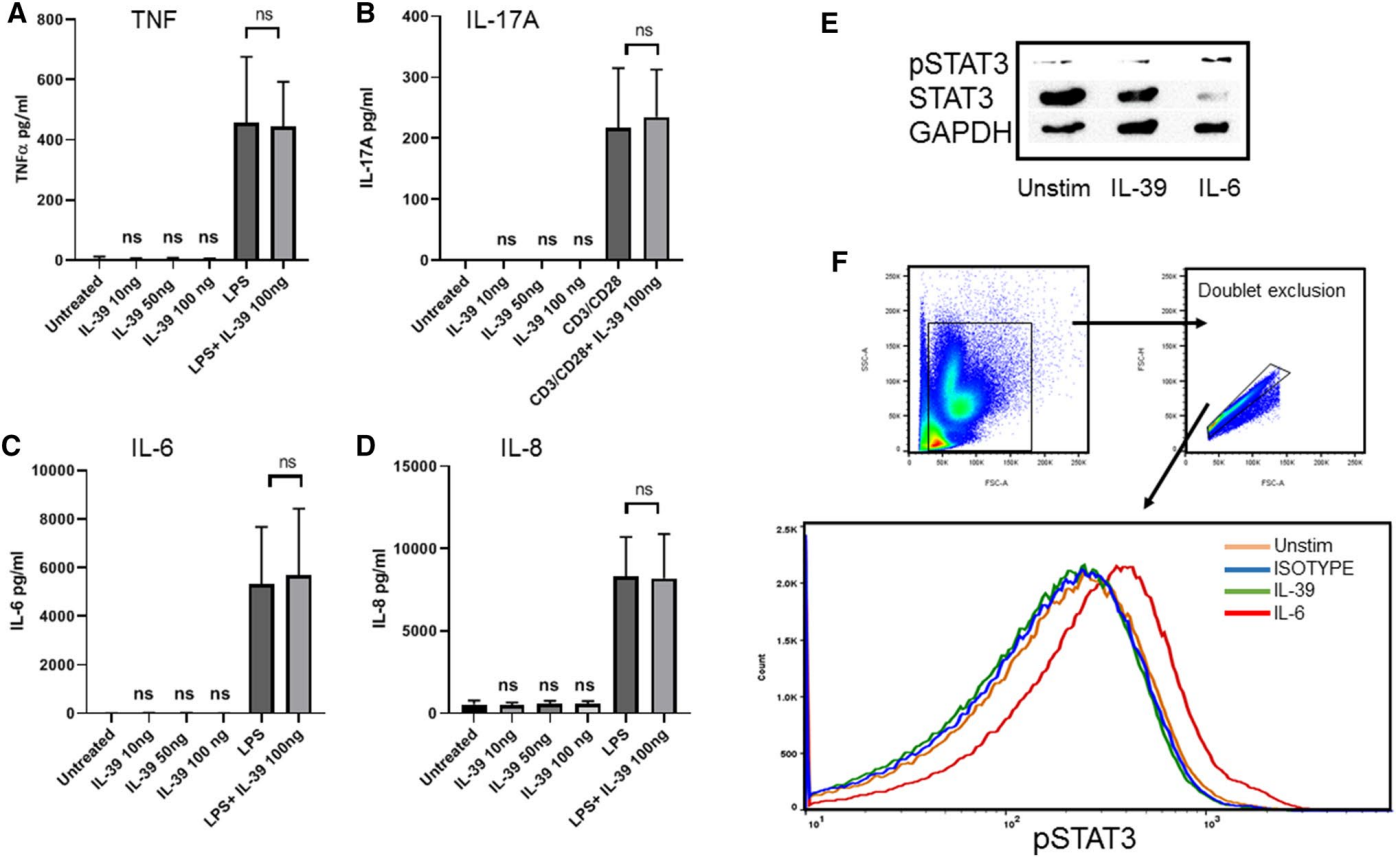
IL-39 shows no activity in human leukocytes. Human leukocytes were stimulated with IL-39 and or LPS, and secreted concentration of TNF, IL-6, and IL-8 was determined by ELISA (**a**, **c** and **d**). Leukocytes were stimulated with IL-39, with and without anti-CD3/CD28, and secreted concentration of IL-17 was determined by ELISA (**b**) (*n* = 4). Leukocytes were stimulated with IL-39 (100 ng/ml) or IL-6 (20 ng/ml) for 15 min and subsequently probed for phospho STAT3 or GAPDH using western blot method (**e**). Cells were stimulated as before, and subsequently intracellularly stained for phospho-STAT3, and signal measured using flow cytometry (**f**). **a**–**d** Significance reported between treatments and unstimulated (unless stated). Paired *t*-test

Previous research from a murine model suggests that IL-39 primarily acts upon neutrophils, so we decided to stimulate whole leukocytes (typically 60–70% neutrophils) with IL-39 chimera protein (10, 50, and 100 ng/ml) for 48 h. No significant induction in IL-6, IL-8, or TNF was achieved in comparison to untreated cells. As expected LPS stimulation resulted in a statistically significant increase in IL-6, IL-8, and TNF. IL-39 was also found to have no additive effect on LPS induced cytokine production (Fig. 1a, c and d).

As IL-23 drives IL-17 secretion from T cells, we also investigated whether IL-39 may share this ability. Leukocytes stimulated with anti-CD3/C28 secreted IL-17 (mean 217 pg/ml), but IL-39 had no effect on IL-17, either alone or additive (Fig. 1b).

### IL-39 does not induce STAT3 phosphorylation

The previous publications on mouse cells suggest that IL-39 signals through STAT3 [9, 13]. When human leukocytes were stimulated with IL-39, no increase in phospho-STAT3 was detected (Fig. 1e, f). As expected, a positive control with IL-6 stimulation increased phospho STAT3.

## Discussion

IL-39 is cytokine implicated in murine lupus models [9]. As IL-39 shares the common p19 chain with IL-23, hypothetically, p19 blockers could be blocking the activity of this cytokine in man. Whilst our findings do not disprove the existence of IL-39 in humans, they suggest that it should remain theoretical for the time being. The case for the existence of IL-39 in humans based on probability is high. While not all human cytokines exist in mouse, all other confirmed members of the IL-12 family have, thus, far proved to exist in both mice and man; however, data seem stronger for murine p40 homodimer, while it is still debated to play a meaningful pathophysiological role in humans [3, 14]. In our study, an IL-39 chimera protein, consisting of EBI3 and IL-23p19 chains linked by a disulphide bond, was unable to stimulate human leukocytes. Furthermore, IL-39 also showed no signaling potential via STAT3 phosphorylation.

Whilst the chimera nature of the protein may not mirror the true structure of the heterodimer, the same protein does have reported activity in mouse splenocytes (R&D systems). The corresponding receptor complex for IL-39 (IL-23R/gp130) has also been shown to be active on murine cells and results in STAT3 activation; however, this has not been confirmed in humans [13]. However, in mice, it has been reported that EBI3 shows activity as a monomer to signal and stimulate cells, meaning that it is plausible that the EBI3 subunit of the heterodimer could be solely responsible for functional effects seen. It is known, however, in human that EBI3 is not secreted as a monomer [15]. A recent report into combinatorial potential of IL-12 chain pairs in human cells was able to detect all other IL-12 family cytokines (IL-12, IL-23, IL-27, and IL-35) but not IL-39, which, along with our study, raises questions over the existence of this cytokine in man [12]. In conclusion, our study fails to confirm a functional response to IL-39 in man, suggesting that this chain pairing may only play a role in murine immune responses.
